# A novel function for α-synuclein as a regulator of NCK2 in olfactory bulb: implications for its role in olfaction

**DOI:** 10.1186/s13578-024-01313-6

**Published:** 2024-11-14

**Authors:** Jing Ren, Chao Wu, Mengxia Zeng, Mingqin Qu, Ge Gao, Ning Chen, Jingjing Yue, Yuwen Jiang, Tongfei Zhao, Na Xiang, Fangang Meng, Ling-ling Lu

**Affiliations:** 1 Department of Neurobiology, Capital Medical University, 10 You’an Men Wai, Xitoutiao, Beijing, 100069 China; 2 Key Laboratory for Neurodegenerative Diseases of the Ministry of Education, Beijing, 100069 China; 3 Department of Functional Neurosurgery, Beijing Tiantan Hospital, Capital Medical University, Beijing, 100070 China; 4 Department of Functional Neurosurgery, Beijing Neurosurgical Institute, Capital Medical University, Beijing, 100070 China

**Keywords:** Parkinson’s disease, α-synuclein, Ephs, NCK2, Synucleinopathies

## Abstract

To investigate physiological function of α-synuclein is important for understanding its pathophysiological mechanism in synucleinopathies including Parkinson’s disease. Employing knockout mice, we found that Snac/α-synuclein deletion induced aberrant projection of olfactory sensory neurons and hyposmia. We identified 9 axon guidance associated differentially expressed proteins using iTRAQ based Liquid Chromatograph Mass Spectrometer. NCK2 is most significantly down-regulated protein among them. We further found that either α-synuclein deletion or NCK2 deficiency induced Eph A4 inactivation. Re-expressing Snac/α-synuclein in its knockout neurons reversed the down-regulation of NCK2, as well as the inactivation of EphA4. Overexpression of Snac/α-synuclein in α-synuclein deleted mice reversed the down-regulation of NCK2 and pEphA4, and improved the olfactory impairment of mice. Correlation analysis showed that there is a significant correlation between the protein level of α-synuclein, NCK2, and pEphA4, respectively. Nonetheless, immunoprecipitation analysis showed that NCK2 was associated with both EphA4 and Rho A, suggesting that NCK2 as a scaffolding protein to modulate Eph A4/Rho A pathway. Moreover, Rho A activity was significantly lower in α-synuclein deficient mice. Thus, α-synuclein regulates olfactory neurons projection through NCK2 dependent EphA4/Rho A pathway. Malfunction of α-synuclein because of deletion may cause aberrant olfactory neurons projection. This extended our knowledge of α-synuclein functions, which may explain why olfaction is usually impaired in some synucleinopathies.

**Supplementary Information:**

The online version contains supplementary material available at 10.1186/s13578-024-01313-6.

## Introduction

Parkinson’s disease (PD) is one of the most common neurodegenerative disorders. Its prevalence is 0.5%–1% among persons of 65–69 years old and 1%–3% among those over the age of 80 years [1]. Although the exact etiology remains unclear, it was believed that aging, genetic and environmental factors play combined roles in its pathogenesis [2, 3]. Study of genes linked to familial Parkinsonism/PD yield critical insights into mechanisms shared by sporadic and familial disease.

SNAC/α-synuclein (α-syn) is the first genetic mutation identified in a Italian heritable PD family. It is responsible for an autosomal-dominant form of PD. α-syn is a small, well-conserved protein that is expressed in many tissues and cell types [4–6]; its main protein domains comprise an amphipathic region, non-amyloid-b component (NAC) domain and an acidic tail [7–9]. Single point mutations in SNAC/α-syn will lead to early onset PD [10–15]. Moreover, triplication of the gene locus [16, 17] and its polymorphisms are also associated with high risk of PD [18, 19]**.** α-syn is not only associated with familial Parkinsonism, it also has been shown to have a central pathogenic role in sporadic PD [20, 21]. Accumulated α-syn is reported to be the main component of Lewy bodies (LBs), the hallmark of PD. Hence, α-syn is the most potent culprit underlying PD. However, both pathogenic mechanisms underlying α-syn with PD and its physiological function remains unclear.

As a neurodegenerative movement disorder, PD is commonly characterized by motor symptoms such as resting tremor, rigidity, akinesia, bradykinesia, and postural instability [22, 23], due to the loss of Nigral dopaminergic neurons and a decrease in the dopamine (DA) contents of the caudate-putamen structures [24]. However, the onset of PD is considered to commence at least 20 years prior to detectable motor deficits. This period is referred to as the prodromal stage when a variety of non-motor symptoms such as olfactory dysfunction, sleep disturbances, obesity and depression etc. can be observed. Olfactory dysfunction is one of the prodromal symptoms and precede motor abnormalities by 20 years [25, 26]. The reported prevalence of olfactory impairment in PD range from 45 to 90%. Nonetheless, Braak and colleagues reported that α-syn pathology was observed in olfactory bulb (OB) in very early stage of PD. In his theory, OB, as one of sensory organs opening to the outside, is susceptible to toxic environment and easy to deteriorate. An external stimuli such as pesticide rotenone and herbicide Paraquat are transmitted through the nasal mucosa to the OB, causing α-syn pathology within the OB [27, 28]. Hence, OB is one of the original sites of α-syn pathology. Then study the physiological function of α-syn in olfactory system will be helpful to understand the onset and progress of PD.

Here, we use Snac/α-syn knockout (Snac ^−/−^, KO) mice and its wild-type (Snac ^+/+^, WT) littermate as a control to study the influence of α-syn deficiency on mice olfaction. The Snac ^−/−^ mice exhibited aberrant projection of olfactory sensory neurons (OSNs) and olfactory dysfunction accordingly. NCK2, identified by proteomics sequencing, and its downstream Eph A4/Rho A were shown to be associated with this α-syn efficiency-induced aberrant projection.

## Materials and methods

### Animals

All the animal experiments were conducted in accordance with the National Institutes of Health guidelines on the care and use of laboratory animals, and were approved by the Animal Care Committee of Capital Medical University. We used male Snac KO mice and their male wild-type (WT) littermates for the present study. Snac KO and WT mice were group housed in a room with a 12 h light/dark cycle (lights on at 7:00 a.m.), with access to food and water ad libitum. Room temperature was kept at 23 ± 2 °C. For α-syn rescue experiments, adeno-associated virus carried Snca (AAV-Snac) was injected into olfactory bulb of Snac KO mice. KO mice injected with AAV without Snac were used as negative control. Olfaction behavior test were done 6 months later.

### Western blot

Equal amounts of protein (100 μg) were separated by 10% sodium dodecyl sulfate polyacrylamide gel electrophoresis and then transferred to a PVDF membrane (Pall Co., Taiwan) that was blocked in 5% non-fat milk for 1 h at room temperature and incubated overnight with primary antibodies against the following proteins: α-synuclein (1:1000, BD, NJ, USA), NCK2 (1:1000, Sigma-Aldrich, MO, USA), pEphA1 (1:500, Abcam, Cambridge, UK), pEphA2 (1:500, Abcam, Cambridge, UK), pEphA3(1:500, Abcam, Cambridge, UK), pEphA4 (1:500, Abcam, Cambridge, UK), pEphA5 (1:500, Abcam, Cambridge, UK), pEph B1/B2 (1:500, Abcam, Cambridge, UK) and β-actin (1:1000, Sigma-Aldrich, MO, USA). Immuno-reactivity was detected with Fluorescence-conjugated anti-mouse or -rabbit antibodies (1:10,000; Sigma-Aldrich, MO, USA).

### Immunohistochemistory

Mice were sacrificed and brains were fixed with 4% paraformaldehyde and sectioned at a 50-μm thickness. The staining was carried out on free-floating sections. Sections were rinsed in PBS and incubated in 3% H_2_O_2_ for 20 min to block the endogenous peroxidase activity. After washing in PBS, sections were incubated in blocking serum (10% goat serum and 0.1% Triton X-100 in PBS) for 30 min, followed by incubation in anti-α-synuclein antibody solution (1:1000, BD, NJ, USA) or olfr1507 (1: 1000, Thermo Fisher, MA, USA) for 24 h at 4 °C. Then, the sections were incubated with Alexa 594-conjugated goat anti-mouse IgG (1: 1000, Cell Signaling Technology, MA, USA) for 2 h at room temperature. Images were captured by a Leica confocal microscope (Leica Microsystems, Wetzlar, Germany).

### Quantitative real time PCR

Total RNA was isolated from the OB of α-syn/Snac KO or its WT littermate control using the RNeasy Mini kit (Qiagen, Düsseldorf, Germany). 5 μg RNA were reverse-transcribed in a reaction containing 1 μl dNTP (10 mmol/l; Invitrogen), 0.5 μg oligo (dT) primer, with 50 U Superscript RNase reverse transcriptase (Invitrogen, CA, USA) for 50 min at 42 °C. Forward and reverse primer sequences for PCR were as follows: β-actin, 5′-ACC ACC ATG TAC CCA GGC ATT-3′ and 5′-CCA CAC AGA GTA CTT GCG CTC A-3′; *NCK2*, 5'-GTCATAGCCAAGTGGGACTACA-3'* a*nd 5'-GCACGTAGCCTGTCCTGTT-3'. The reaction was carried out on 7500 real-time PCR systems (Applied Biosystems, Foster City, CA, USA) using the default thermal cycling conditions (2 min at 50 °C plus 10 min at 95 °C for the hot start; and 40 cycles of 15 s at 95 °C plus 1 min at 60 °C).

### iTRAQ based liquid chromatograph mass spectrometer (LC–MS)

OB tissue were taken from 8-week-old male α-syn/Snac KO mice and their male wild-type littermates. Two biological replicates of each group were prepared for the proteomics experiments. Briefly, the total protein of OB was grinded and dissolved in lysis buffer [9 M Urea, 4% CHAPS, 1%DTT, 1%IPG buffer (GE Healthcare, IL, USA)]. The mix was incubated at 30 °C for 1 h and centrifuged at 15,000*g* for 15 min at room temperature. The supernatant was collected and quantified by the Bradford method.

100 µg of protein of each sample was dissolved in a dissolution buffer (AB Sciex, Foster City, CA, USA). After being reduced, alkylated and trypsin-digested, the samples were labeled following the manufacturer's instructions for the iTRAQ Reagents 8-plex kit (AB Sciex, Foster City, CA, USA). Samples were each labeled with iTRAQ reagents with molecular masses of 113, 114, 115, and 116 Da, respectively. After labeling, all samples were pooled and purified using a strong cation exchange chromatography (SCX) column by Agilent 1200 HPLC (Agilent, CA, USA) and separated by liquid chromatography (LC) using a Eksigent nanoLC-Ultra 2D system (AB SCIEX, CA, USA). The LC fractions were analyzed using a Triple TOF 5600 mass spectrometer (AB SCIEX, CA, USA). Mass spectrometer data acquisition was performed with a Triple TOF 5600 System (AB SCIEX, CA, USA) fitted with a Nanospray III source (AB SCIEX, CA, USA) and a pulled quartz tip as the emitter. Data were acquired using an ion spray voltage of 2.5 kV, curtain gas of 30 PSI, nebulizer gas of 5 PSI, and an interface heater temperature of 150 °C. For information dependent acquisition (IDA), survey scans were acquired in 250 ms and as many as 35 product ion scans were collected if they exceeded a threshold of 150 counts per second (counts/s) with a 2^+^ to 5^+^ charge-state. The total cycle time was fixed to 2.5 s. A rolling collision energy setting was applied to all precursor ions for collision-induced dissociation (CID). Dynamic exclusion was set for ½ of peak width (18 s), and the precursor was then refreshed off the exclusion list.

The iTRAQ data were processed with Protein Pilot Software v4.0 against the *A. japonicus* database using the Paragon algorithm. Protein identification was performed with the search option of emphasis on biological modifications. The database search parameters were the following: the instrument was Triple TOF 5600, iTRAQ quantification, cysteine modified with iodoacetamide; biological modifications were selected as ID focus, trypsin digestion. For false discovery rate (FDR) calculation, an automatic decoy database search strategy was employed to estimate FDR using the PSPEP (Proteomics System Performance Evaluation Pipeline Software, integrated in the ProteinPilot Software). The FDR was calculated as the number of false positive matches divided by the number of total matches. Then, the iTRAQ was chosen for protein quantification with unique peptides during the search, and peptides with global FDR values from fit less than 1% were considered for further analysis. Within each iTRAQ run, differentially expressed proteins were determined based on the ratios of differently labeled proteins and p-values provided by Protein Pilot; the p-values were generated by Protein Pilot using the peptides used to quantitate the respective protein. Finally, for differential expression analysis, fold change was calculated as the average ratio of 113/ 114, 113/116, 115/114 and 115/116. Proteins with a fold change of > 1.5 or < 0.67 and p < 0.05 were considered to be significantly differentially expressed.

### Rho A activation assay

Assays for the active form of Rho A was performed, as described previously [45]. Briefly, primary neurons were seeded on 24 mm dishes and cells were cultured to approximately 85–90% confluence. Cells were lysed in Mg2 + Lysis/Wash Buffer containing 25 mM HEPES, pH 7.5, 150 mM NaCl, 1% Igepal CA-630, 10 mM MgCl2, 1 mM EDTA and 2% glycerol, supplemented with 10 μg/mL aprotinin and 10 μg/mL leupeptin and subjected to Rho A pull-down assays with rhotekin RBD agarose (Millipore, MA, USA), which would bind and detect active RhoA. GTP bound RhoA was separated on SDS–polyacrylamide gel electrophoresis gels and subjected to Western blot analysis with the RhoA antibody.

### Quantification and statistical analysis

Data are expressed as mean ± SD. The sample size (n) represents biological replicates. Student’s t test was used for comparison of two group averages. When there were more than two groups, one-way ANOVA Tukey − Kramer multiple comparisons Bonferroni post hoc test was performed. All the bioinformatics were analyzed or confirmed by bioinformaticians. iTRAQ based LC–MS analysis was performed in a double-blinded manner. A P value < 0.05 was considered statistically significant.

## Results

### α-syn was abundantly expressed in both olfactory epithelium and OB

**Fig. 1:**
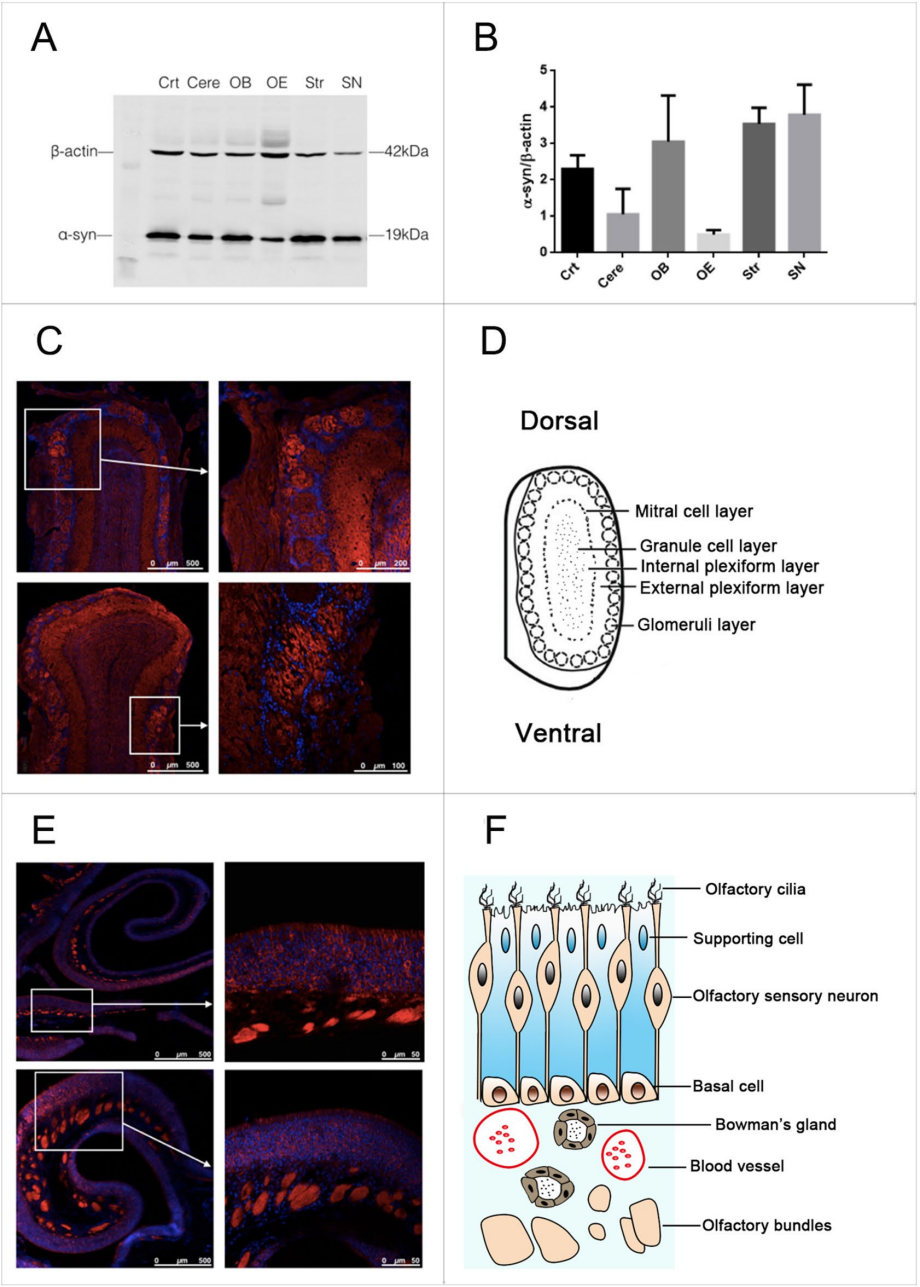
α-syn was abundant in both olfactory epithelium and OB. **A**, **B** α-syn was expressed in different brain regions. α-syn protein level was investigated in various brain regions. It was abundant in olfactory system, especially in OB. Data are expressed as mean ± SD, N = 3. **C** Immunofluorescence staining to show α-syn expression pattern in OB. α-syn was mainly located on olfactory glomeruli and outer plexiform layer of OB. **D** Schematic diagram of OB. **E** Immunofluorescence staining to show α-syn expression pattern in OE. α-syn is abundant in the soma of OSNs and olfactory bundles of OE. F Schematic diagram of OE

In order to investigate α-syn expression pattern in the brain, western blot was used to evaluate α-syn protein level in different brain regions eg. cortex (Crt), cerebellum (Cere), OB, olfactory epithelium (OE), striatum (Str) and substantia nigra (SN). As was shown in Fig. 1, α-syn was abundant in olfactory system, especially in OB. Immunofluorescence staining showed that α-syn is specifically expressed in the soma of olfactory sensory neurons (OSNs) and the olfactory bundles of OE. α-syn was was also observed in olfactory glomeruli and outer plexiform layer of OB (Fig. 1).

### α-syn deficiency induced aberrant projection of OSNs and resulted in hyposmia

**Fig. 2:**
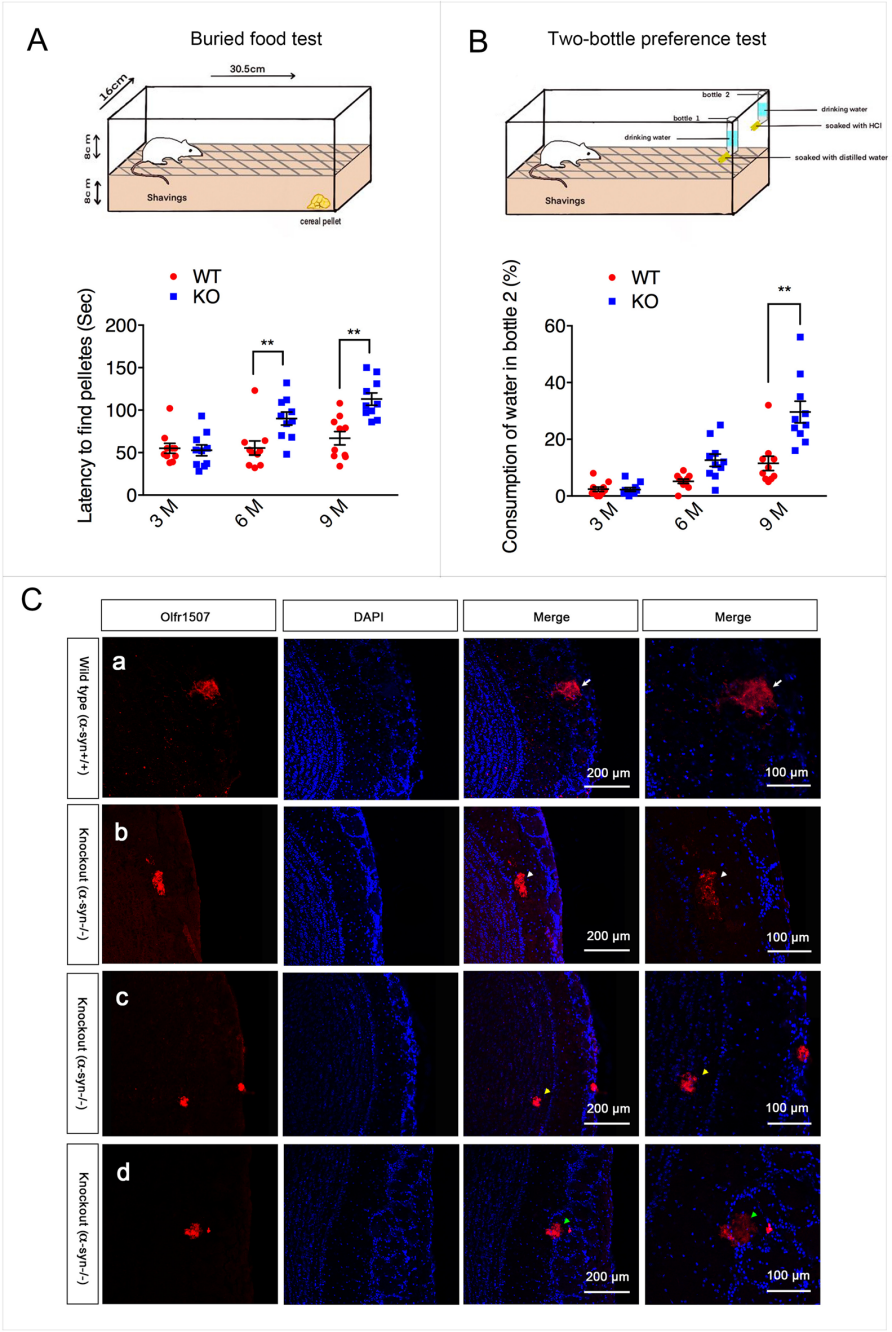
α-syn deficiency induced olfaction impairment and aberrant projection of olfactory neurons. **A** Statistical results of buried food pellet test. Data are expressed as the mean ± SEM.**p < 0.01, n = 10. **B** Statistical results of two-bottle preference test. Data are expressed as the mean ± SEM. **p < 0.01, n = 10. **C** Immunofluorescence staining using an antibody against the Olfr1507 revealed that Olfr1507-expressing OSNs axon fibers converge to one specific glomerulus in each half bulb in wild type mice(WT, α-Syn^+/+^) as indicated by the white arrow. In α-syn null mice (KO, α-Syn^−/−^), Olfr1507 -expressing OSNs axons projected aberrantly. Some Olfr1507 positive axons projected to external plexiform layer as indicated by the white arrowhead. Some projected to the inner plexiform layer as indicated by the yellow arrowhead. And some projected out of the glomerulus although it still locates in the glomerulus layer as indicated by the green arrowhead

As we know, the mapping of OSNs onto glomeruli astonishingly precise. Each glomerulus receives axons from a large number of OSNs that express the same olfactory receptor (OR). So doing immunofluorescence staining of OB with one specific OR antibody, only one positive glomerulus in each bulb will be detected. Olfr1507, one kind of OR, antibody was used to evaluate the axon projection in α-syn/Snac KO mice in the present study. Of note, Olfr1507 positive axons projected aberrantly in α-syn deficient mice. Some Olfr1507 positive axons projected to external plexiform layer. Some projected to the inner plexiform layer. And some projected out of the glomerulus although it still locates in the glomerulus layer (Fig. 2). Buried pellet test and two-bottle preference test were used to evaluate the olfactory function of α-syn deficient (KO) mice. In the buried food test, KO mice spent more time to locate the buried pellet compared with WT mice by 6 months (P < 0.01, Fig. 2). In the two-bottle preference test, there were no significant differences between the strains in water intake before 3 months age. The littermate KO mice started to consume water from bottle treated with 0.7% hydrochloride acid at 6 months old and kept increasing at 9 months old, however, control animals still displayed a strong preference for water from non-treated bottle. (P < 0.05 for 6 months and P < 0.01 for 9 months, Fig. 2).

### 9 axon guidance associated proteins was identified by iTraq based LC–MS

In KO mice, Olfr1507 positive axons project aberrantly which indicates that α-syn played an important role in olfactory axons guidance. To find out the molecules involved in this process, iTraq based LC–MS was carried out to compare the differentially expressed proteins between KO mice and its litter mate control.

The screening criteria of reliable proteins: Unique peptide ≥ 1, remove invalid values and the anti-library data, and screening significant differentially expressed protein based on the reliable proteins. The screening criteria of differentially expressed protein: Fold change is 1.5. Changes greater than 1.5 or less than 0.67 are considered to be differentially expressed proteins. Screening results statistics of differentially expressed proteins were as follows:

1. Total number of all proteins: 5489 pcs.
2. Total number of reliable proteins: 4936 pcs.
3. Number of differentially expressed proteins in Dynamics: 278 pcs.
4. The number of differentially expressed proteins in Snac KO mice and its litter mate control: 188 pcs.

**Fig. 3:**
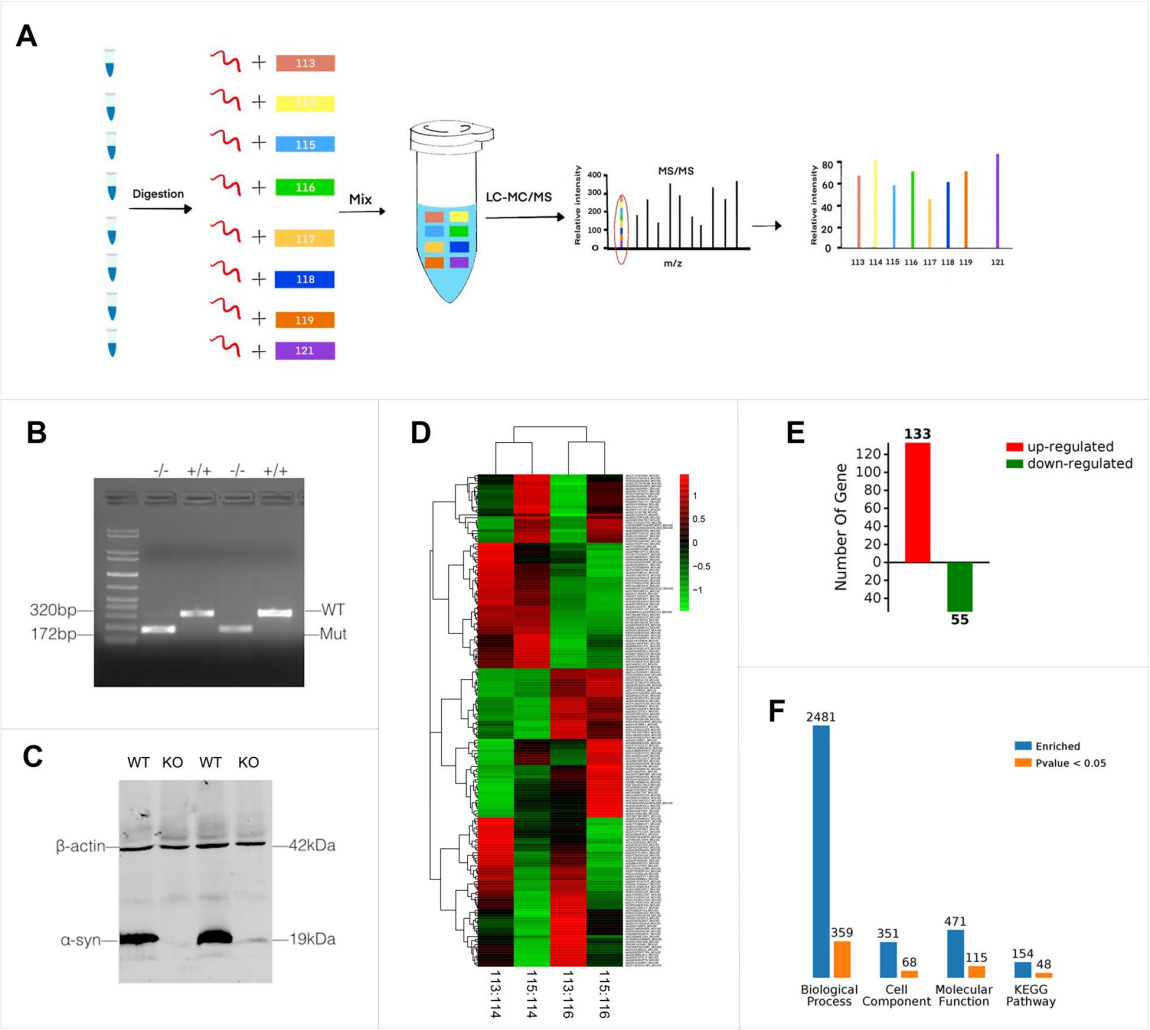
Identification of differentially expressed proteins in α-syn null mice by iTRAQ based LC–MS. **A** Flow chart for differentially expressed proteins identification. **B**, **C** Genotyping and western blotting to confirm the mice genotype. **D** The heatmap for the differentially expressed proteins. In the heatmap, red color represents up-regulated differentially expressed proteins and the green color represents down-regulated differentially expressed proteins. **E** statistical summary for differentially expressed proteins*.* A total of 188 proteins, can be seen in the figure in the difference comparison group. There are 133 pcs of up-regulation (red) of the protein and 55 pcs of down-regulation (green) of the protein. **F** All the differences in protein GO enrichment (BP, CC, MF) and pathway KEGG enrichment results and significant (p < 0.05) number were summarized. The blue column represents the total number of enrichment, orange column represents a significant number of enrichment. A protein is usually involved in a number of functions or pathways, so the total number of GO enrichment and pathway KEGG enrichment results will be far greater than the number of differentially expressed proteins. The significant function or the pathway means that the difference of the enrichment to the function or the pathway is significant

Among all above 188 differentially expressed proteins, 133 were up-regulated and 55 were down-regulated (Fig. 3). Further analysis showed that 9 of differentially expressed proteins were associated with axon guidance (Table 1). The expression level of these 9 differentially expressed proteins was first verified by quantative real-time RT-PCR. The results showed that 5 of them including Cdh4, Cldn5, Dscam, Eif2b2, Pllp, Snapin were up-regulated and 4 of them including Snapin, Spag1, Stk11 and NCK2 were down-regulated. NCK2 was one of the most significant one that down-regulated by α-syn deletion.

**Table 1:** Axon guidance associated differentially expressed proteins

NO	Protein	Full name	Ratio
1	Cdh4	Cadherin-4	1.66
2	Cldn5	Claudin-5	2.21
3	Dscam	Down syndrome cell adhesion molecule homolog	1.86
4	Eif2b2	Translation initiation factor eIF-2B subunit beta	1.51
5	Pllp	Plasmolipin	1.57
6	Snapin	SNARE-associated protein Snapin	0.64
7	Spag1	Sperm-associated antigen 1	0.66
8	Stk11	Serine/threonine-protein kinase	0.52
9	NCK2	Cytoplasmic protein NCK2	0.58

### α-syn modulated NCK2 protein expression level

**Fig. 4:**
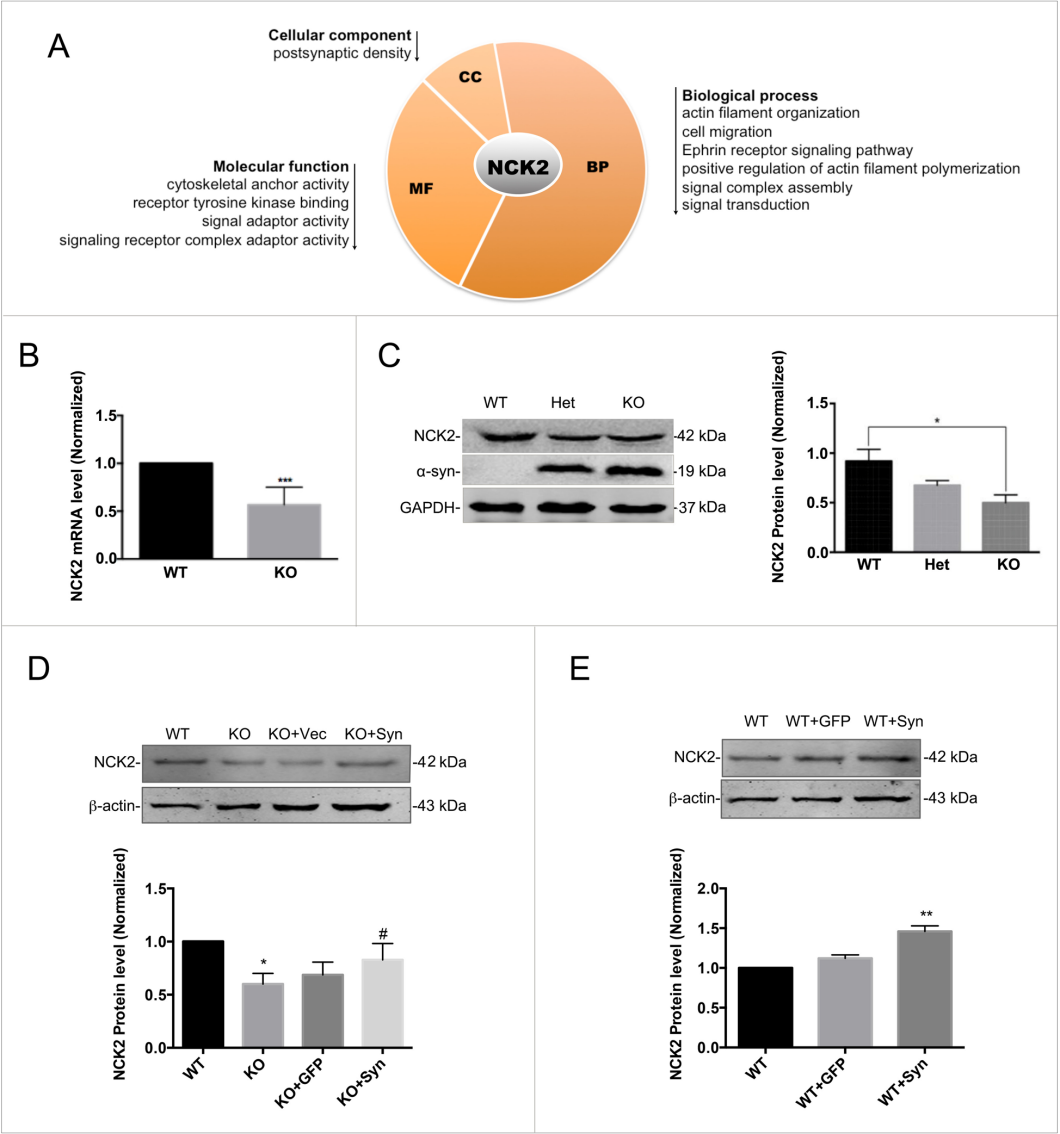
α-syn regulated the protein expression level of NCK2. **A** Bioinformatics analysis for the most down-regulated protein, NCK2 showed that NCK2 was mainly located in the postsynaptic density. Its function involved in cytoskeletal anchor, signal adaptor, cell migration etc. **B** NCK2 down-regulation was further confirmed in mRNA level by real-time RT-PCR. Data are expressed as mean ± SD. ***p < 0.001 compared to WT. C NCK2 down-regulation was further confirmed in protein level by western-blot. Data are expressed as mean ± SD. *p < 0.05 compared to WT. D α-syn rescue experiment. NCK2 down-regulation was reversed by overexpression of α-syn in the α-syn^−/−^ primary cultured neurons. Data are expressed as mean ± SD. *p < 0.05, compared to WT; #p < 0.05 compared to KO; n = 3. E α-syn overexpression also induced NCK2 up-regulation. Data are expressed as mean ± SD. **p < 0.01 compared to WT; n = 3

Cytoplasmic protein NCK2, one of the most significant down-regulated proteins, is shown to bind and recruit various proteins involved in the regulation of receptor tyrosine kinases family (RTKs). Bioinformatic analysis for NCK2 showed that it takes part in the following biological process, eg. actin filament organization, cell migration, Ephrin receptor signaling pathway, positive regulation of actin filament polymerization, signal complex assembly and signal transduction. The first 4 process are directly associated with axon guidance and the last two process are also related to the axon guidance indirectly. The following molecular function analysis showed that it exhibited cytoskeletal anchor activity, receptor tyrosine kinase binding function, signal adaptor and signaling receptor complex adaptor activity. All the above molecular functions implicated that it takes part in axonal guiding. Cellular component analysis indicated its subcellular localization of post-synapsis are consisting with its function-axon guidance (Fig. 4A).

As shown above, NCK2 is also one of the most significantly down-regulated proteins in α-syn/Snac KO mice in the present study (Table 1). So we chose NCK2 as the following research focus. The down-regulation of NCK2 was further confirmed both in mRNA level and protein level by real-time RT-PCR and western-blotting (Fig. 4B, C). Nonetheless, the NCK2 down-regulation was reversed by overexpression of α-syn in the SNAC^−/−^ primary cultured neurons (Fig. 4D). On the other side, α-syn overexpression also induced NCK2 up-regulation in protein level (Fig. 4E).

### NCK2-EphA4 was involved in the mis-guidance of olfactory sensory neurons

**Fig. 5:**
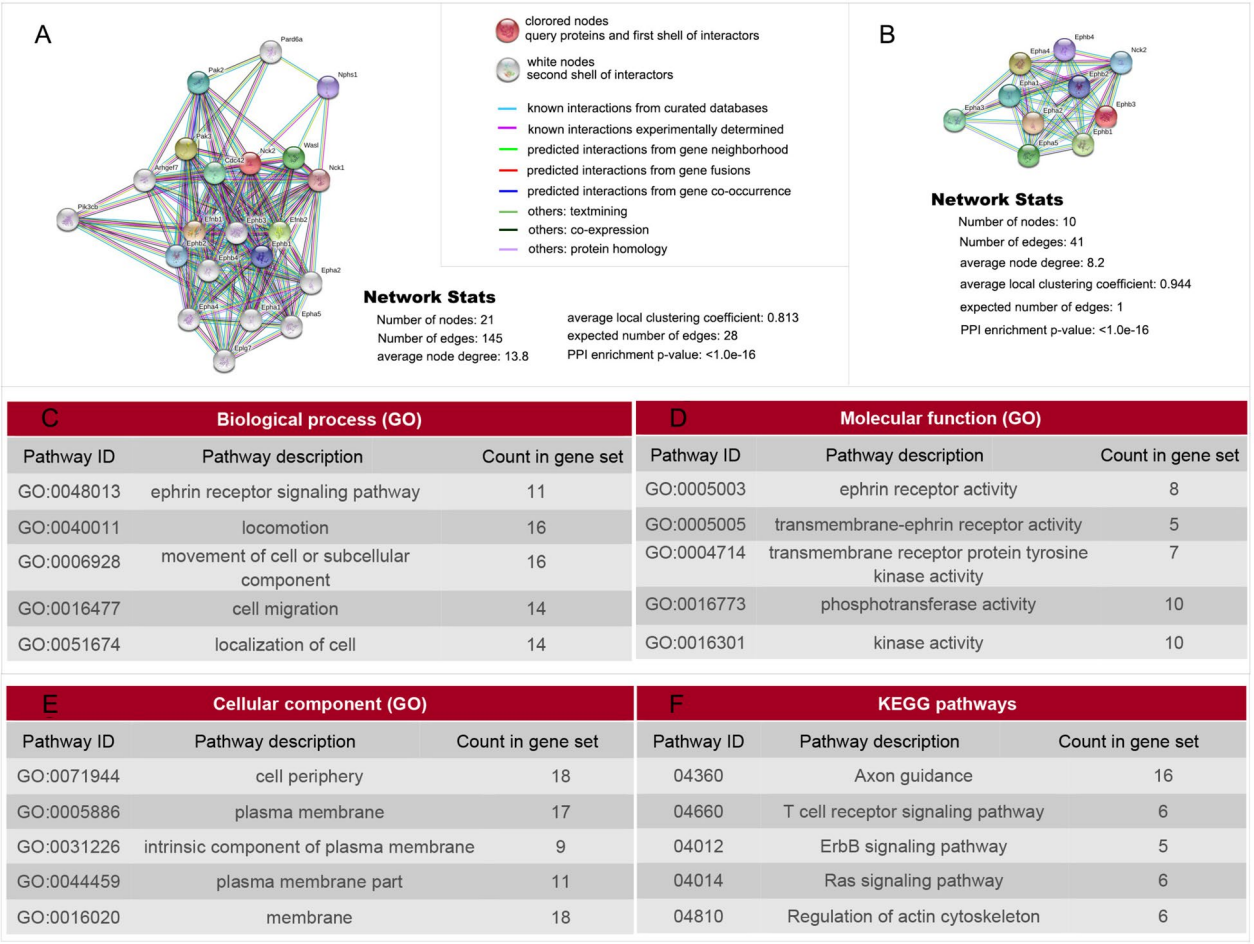
Bioinformatics analysis showed that 21 proteins were enriched in PPI network with NCK2. **A** PPI network with NCK2. Bioinformatics analysis showed that 21 proteins were enriched in PPI network with NCK2. Biological analysis for these 21 proteins showed that 11 of them were enriched in Ephrin receptor signaling pathway. **B** Further analysis on the association of NCK2 and all Ephrin receptors. And indirect or predicted interactions existed in NCK2 and Eph A1-A5, EphB1-B4. **C** GO analysis showed that all these 21 proteins involves the following biological process: Ephrin receptor signaling pathway, locomotion, movement of cell or subcellular component, cell migration, localization of cell. **D** GO analysis showed that all these 21 proteins involves the following molecular function: Ephrin receptor activity, trans-membrane receptor protein tyrosine kinase activity etc. **E** GO analysis showed that all these 21 proteins were localized on cell periphery, plasma membrane. F KEGG analysis showed that all these proteins involves axon guidance etc.

Bioinformatics analysis showed that 20 proteins eg. EphA1, EphA2, EphaA4, Eph A5, EphB1, EphB2, EphB3, EphB4, EfnB1,EfnB2, Eplg7, Pik3cb, NCK1, wasl, cdc42, pak3, Arhgef7, Nphs1, pak2,pard6a, were enriched in Protein–Protein Interaction (PPI) network with NCK2 (p < 1.0e−16) (Fig. 5A). Analysis for the Network Analysis PPI between NCK2 and Ephs family showed that NCK2 associated with all of the Ephs family members. Most of them were known interactions experimentally determined (Fig. 5B). Kyoto Encyclopedia of Genes and Genomes (KEGG) analysis for these 20 proteins suggested that 16 were enriched in axon guidance pathway (Fig. 5F). Biological process (BP) analysis showed that 11 of them were enriched in Ephrin receptor signaling pathway, one of the important anxon guidance pathways. 16 out of 20 proteins were involved in movement of cell or subcellular component. 14 were involved in cell migration and 14 were involved in localization of cell. All these biological process are associated with axon guidance directly or indirectly (Fig. 5C). Molecular function (MF) analysis showed that 8 out of 20 proteins exhibited Ephrin receptor activity. And some of the proteins take part in trans-membrane Ephrin receptor activity, trans-membrane receptor protein tyrosine kinase activity, phospho-transferase activity, kinase activity which are associated with axon guidance directly or indirectly (Fig. 5D). Cellular component (CC) analysis showed that most of the proteins were located on membrane which are also consistent with their axon guiding function (Fig. 5E). Taken together, both gene ontology (GO) including BP, MF and CC analysis, and KEGG analysis implicated that NCK2 participated axon guidance via Ephrin receptor signaling pathway.

**Fig. 6:**
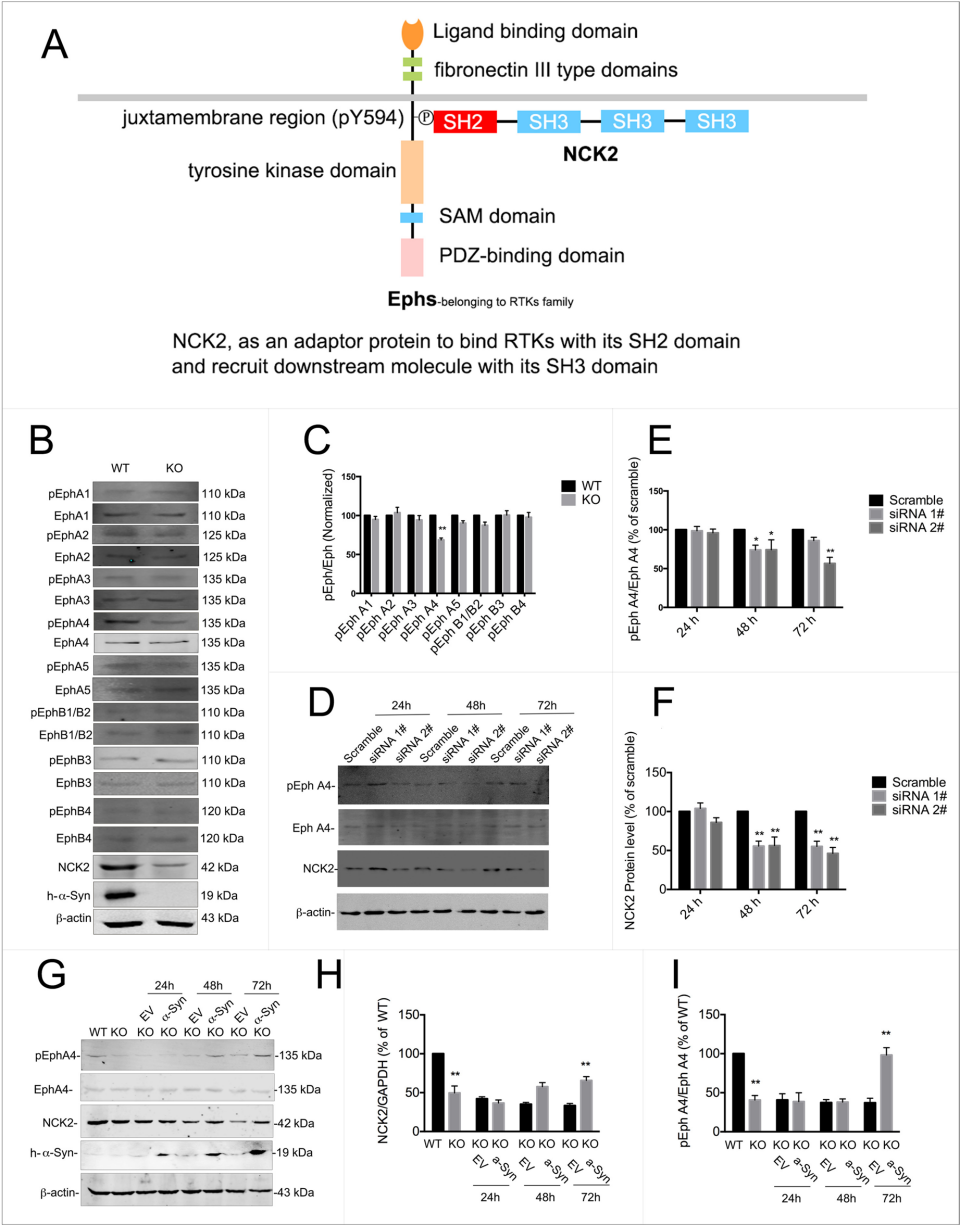
EphA4 was inactivated with α-syn or NCK2 deficiency. **A** A model graph for NCK2 binding Ephs. **B**, **C** All 9 phosphorylated Ephrin receptors were detected by western blot. As shown above, the phosphorylation level of EphA1, EphA2, EphA3, EphA5, EphB1/B2, EphB3 and EhpB4 were not changed. However, phosphorylated Eph A4 level was down-regulated with α-syn deficiency. **D**, **F** Knocking-down of NCK2 also led to down-regulation of phosphorylated Eph A4. **G**–**I** The primary cultured α-syn^−/−^ neurons were transfected with LV-α-syn. NCK2 and phosphorylated EphA4 were down-regulated in α-syn.^−/−^ neurons. The down-regulation of NCK2 and phosphorylated EphA4 were reversed by overexpression of α-syn. Data are expressed as mean ± SD. *p < 0.05, **p < 0.01 compared to WT or scramble, respectively; n = 3

**Fig. 7:**
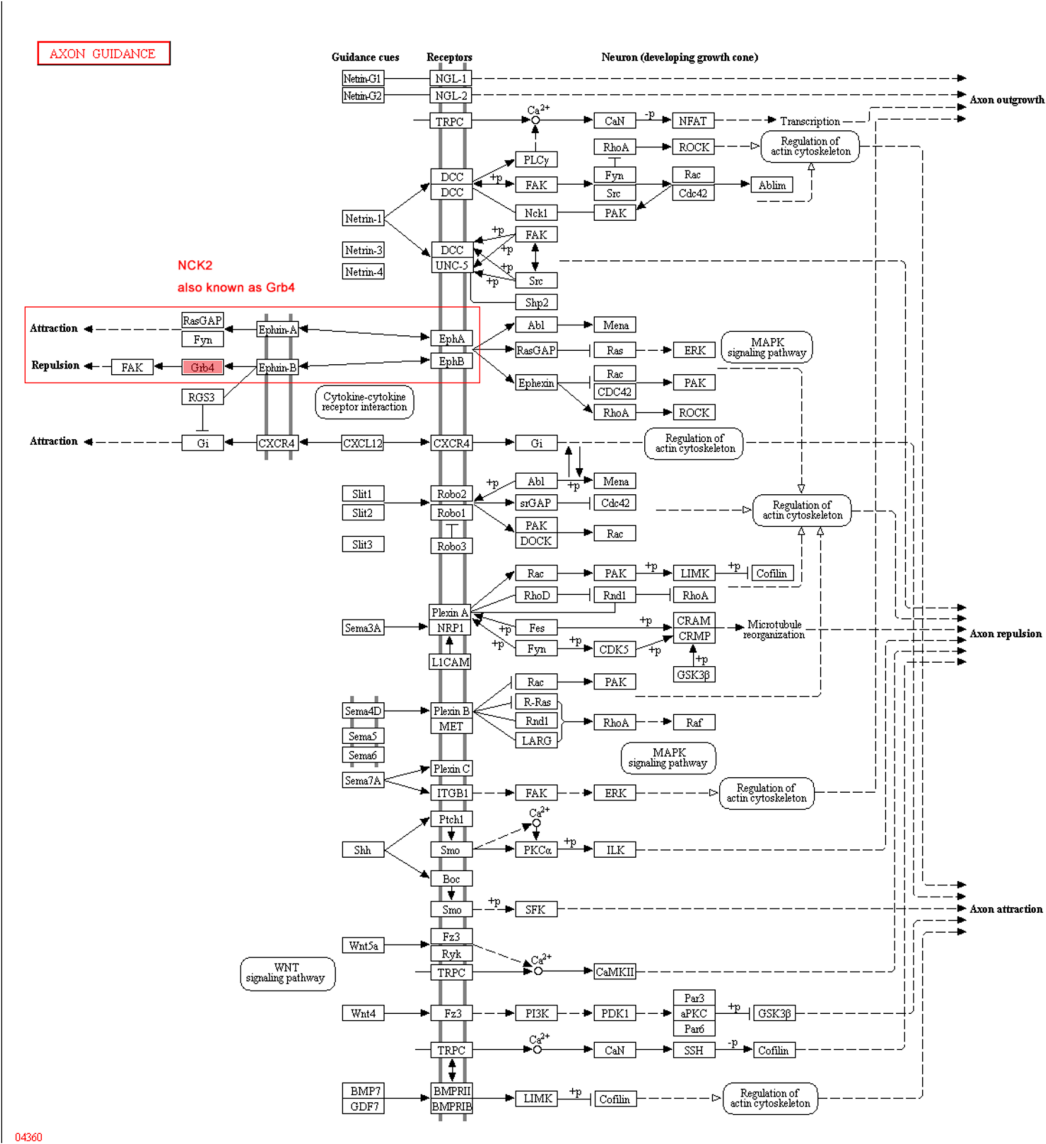
Axon guidance pathway analysis. The axon guidance pathway showed that both Ephrin A/Eph A and Ephrin B/EphB receptor signaling pathway involved in axon guidance, either on repulsion or attraction by cooperation with NCK2

Ephrin receptor signaling pathway, also known as Eph/Ephrin signaling pathway consists of Eph receptors and Ephrin ligands. The Ephs are the largest family of RTKs in vertebrates, and are composed by A- and B-subfamilies. As mentioned above, NCK2 is able to bind and recruit various proteins involved in the regulation of RTKs. And one of the important process that NCK2 participated included ephrin receptor signaling pathway. Nonetheless, NCK2, an adaptor protein consists of one SH2 domain and 3 SH3 domains, can also binds to the tyrosine residues of RTKs via its SH2 domain (Fig. 6A). The axon guidance pathway showed that both Eph A and Eph B receptor signaling pathway involved in axon guidance, either on repulsion or attraction (Fig. 7).

Hence, to find out whether Ephrin receptor signaling pathway involved in the aberrant axon projection of olfactory sensory neurons induced by α-syn deficiency, all 9 Eph receptors in the above NCK2 network were examined in Snac ^−/−^ mice. As shown in Fig. 6B, C, among 9 of Ephs, EphA4 was inactivated which was indicated by its lower phosphorylated level in α-syn deficient mice. While the other 8 Ephs didn’t change with α-syn deficiency. Moreover, knocking-down NCK2 led to EphA4 inactivation as well (Fig. 6D–F).

To further confirm that NCK2-EphA4 was modulated by α-syn, the primary cultured α-syn^−/−^ neurons were transfected with LV-α-syn. NCK2-EphA4 pathway was evaluated by western blot. As shown above, NCK2 and phosphorylated EphA4 were down-regulated in α-syn^−/−^ neurons, (Fig. 6G–I) indicating that NCK2-EphA4 pathway was deactivated. However, the down-regulation of NCK2 and phosphorylated EphA4 were reversed by overexpression of α-syn.

### Overexpression of α-syn reversed the down-regulation of NCK2 and pEphA4, and improved hyposmia of α-syn deficient mice

**Fig. 8:**
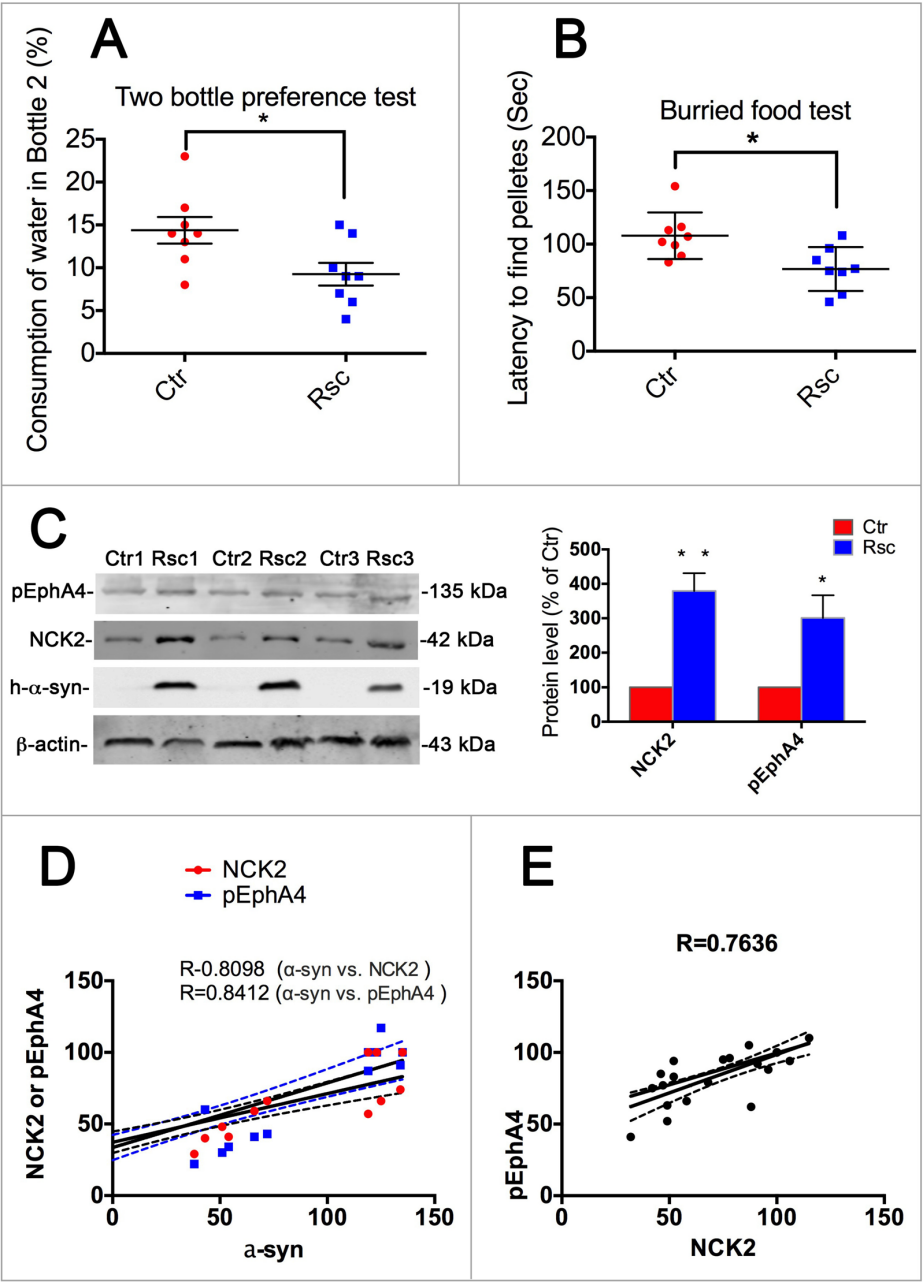
Overexpression of α-syn reversed the down-regulation of NCK2 and pEphA4 and rescued the olfactory dysfunction of α-syn KO mice accordingly. **A**, **B** Two bottle preference test and burried food test. Data are expressed as mean ± SEM. *p < 0.05; n = 8. **C** Western blot for NCK2 and pEphA4. Data are expressed as mean ± SEM. *p < 0.05; n = 3. **D** Correlation analysis among α-syn, NCK2 and pEphA4. R = 0.8098 α-syn vs. NCK2, R = 0.8412 α-syn vs. pEphA4. **E** Correlation analysis between NCK2 and pEphA4. R = 0.7636

We confirmed that α-syn and NCK2 played important roles in modulating Eph A4 activity by knocking-down or overexpressing of these two genes in the above experiments. As is shown above, α-syn and NCK2 were involved in the olfaction impairment in α-syn/Snac KO mice. We further overexpressed α-syn in 4-week-old KO mice to observe whether it could improve the symptoms of olfactory dysfunction in mice (Fig. 8A, B). The results showed that overexpression of α-syn could alleviate olfactory dysfunction in mice. Moreover, the down-regulation of NCK2 and pEphA4 were also reversed (Fig. 8C). We then analyzed the correlation among α-syn, NCK2 and pEphA4. As was shown in Fig. 8D, E, there is a significant correlation between α-syn, NCK2, and pEphA4, respectively,

### NCK2 was associated with EphA4 and NCK2 down-regulation led to lower Rho A activity

**Fig. 9:**
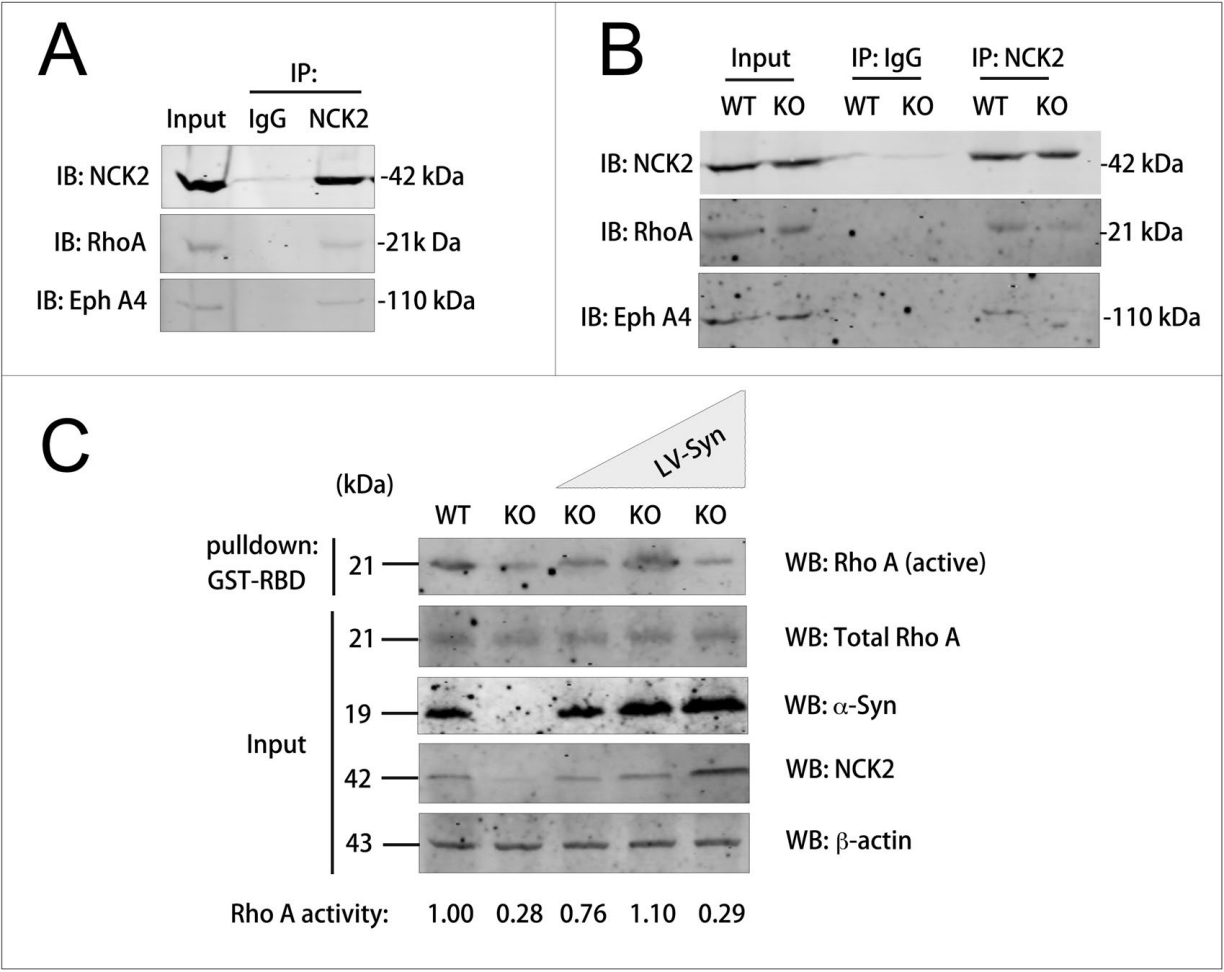
NCK2 were associated with EphA4 and Rho A and acted as an upstream regulator of Rho** A** A NCK2 interacted with EphA4 and Rho A under physiological conditions. Tissue lysates of C57BL/6J mice were incubated with NCK2 antibody or immunoglobulin G (IgG) control for immunoprecipitation and then Western blotted with NCK2, EphA4 and Rho A antibodies. Both EphA4 and Rho A were pulled-down with NCK2 antibody. **B** Cell lysates of primary neurons from Snac ^−/−^ mice (KO) or its littermate WT control (WT) were immunoprecipitated with anti-NCK2 antibodies and Western blotted with EphA4 and Rho A antibodies. Less EphA4 and Rho A were pulled-down in α-syn^−/−^ neurons compared with WT control. **C** Endogenous Rho A activity of primary neurons were examined in the presence of different amounts of NCK2. Lysates of α-syn^−/−^ neurons or α-syn^+/+^ neurons or transiently overexpressing gradually increasing amount of α-syn (illustrated by blue triangle) were used for pull-down with immobilized glutathione S-transferase (GST)–RBD (GST-RBD) of rhotekin and then western blotted with RhoA, NCK2, α-syn and β-actin antibodies. The ratio of active Rho A to total Rho A is normalized to WT control in lane 1 and labeled at the bottom

As shown above (Fig. 6A), it was reported that NCK2, as an adaptor protein, binds to the phosphorylated tyrosine residue of the upstream signal protein eg. RTKs via SH2 domain and binds to the proline rich domain of the downstream signal protein eg. Rho A via SH3 domain. Ephs family and Rho A signaling pathway plays important roles in axon guidance. Hence, to address the molecular mechanism how NCK2 involved in the misguidance of OSNs induced by α-syn deficiency, we investigate the association among NCK2, Eph A4 and Rho A using immunoprecipitation assay. The results showed that NCK2 could preferentially interact with Rho A and Eph A4 (Fig. 9A). Nonetheless, less Eph A4 and Rho A were pulled-down when immunoprecipitated by using NCK2 antibody in KO mice than WT mice (Fig. 9B).

To investigate the downstream signal protein Rho A activity, RBD pull-down assay was employed to determine the active Rho A levels in primary neurons of Snac /α-syn KO mice in the absence of endogenous NCK2, as well as in primary neurons with different overexpression levels of α-syn and NCK2. The level of active RhoA in KO neurons were greatly reduced with lower level of NCK2, increased in neurons overexpressing small dosage of α-syn and NCK2 and decreased to be lower than WT control in neurons with higher expression of α-syn and NCK2 (Fig. 9C). These results suggested that NCK2 acted as an upstream regulator of RhoA and that the role toward Rho A activity can be either positive or negative depending on the relative amount of its protein level. This concentration-dependent manner is a typical biphasic scaffold effect, as similarly shown by studies on BPGAP1 [29].

## Discussion

It was reported that α-syn null mice display striking resistance to MPTP-induced degeneration of Dopaminergic neurons and DA release, and this resistance appears to result from an inability of the toxin to inhibit complex I [30]. But later Abeliovich et al. showed that mice lacking α-syn displayed a reduction in striatal DA and an attenuation of DA-dependent locomotor response to amphetamine [31]. These findings support the hypothesis that α-syn is an essential protein in central nervous system. It plays an important role in many physiological functions in vivo. Up to now, the physiological function of α-syn that was reported include: 1) α-syn functions in synaptic neurotransmitter release by binding to synaptic vesicles and regulating synaptic vesicle pool organization [32, 33]. 2) α-syn, a presynaptic protein that plays a crucial role in dopamine compartmentalization in the striatum, is also involved in the compartmentalization of norepinephrine in the dentate gyrus [34]. 3) α-syn modulates brain glucose metabolism because α-syn deficiency leads to increased glyoxalase I expression and glycation stress [35]. 4) What’s more, it was shown that α-syn/Snac KO mice had decreased striatal dopamine and altered dopamine release, uptake in response to electrical stimuli, which suggests that α-syn may have a role in dopamine neurotransmission [36]. And similarly Connor-Robson et al. reported that stabilization of the striatal DA level depends on the presence of α-syn and cannot be compensated by other family members [37]. α-syn modulates dopaminergic neuron development, DA reuptake and stabilizes striatal DA level [36–38]. 5) α-syn modulates microglial phenotype and neuroinflammation [39, 40]. 6) in Kokhan et al.’s experiment, α-syn/Snac KO mice have cognitive impairments [41]. α-syn/Snac KO mice showed impaired spatial learning and working memory. 7) Using co-immunoprecipitation techniques, Alim et al. found α-syn significantly interacts with tubulin and that α-syn may behave as a potential microtubule-associated protein [42]. Here we report a novel function of a-syn that it is essential for OSNs axon projection. α-syn was highly expressed in both olfactory epithelium and OB. Lacking of α-syn induced aberrant projection of OSNs.

188 differentially expressed proteins resulted from α-syn deficiency were identified by iTraq based LC–MS. 9 of them were associated with axon guidance, including Cdh4, Cldn5, Dscam, Eif2b2, Pllp, Snapin, Spag1, Stk11, and NCK2. These results suggest that Cdh4, Cldn5, Dscam, Eif2b2, Pllp, Snapin, Spag1, Stk11, NCK2 may be involved in the modulation of olfactory neurons projection by α-syn. In the present study, we further confirmed that NCK2 was down-regulated in α-syn^−/−^ mice both in mRNA and protein level. NCK2 belongs to a family of adaptor proteins and NCK2 is located in chromosome 2. The protein contains three SH3 domains and one SH2 domain. The protein has no known catalytic function but has been shown to bind and recruit various proteins involved in the regulation of RTKs. It is through these regulatory activities that this protein is believed to be involved in cytoskeletal reorganization. In the present study, we examined NCK2 targeted receptor protein tyrosine kinases Ephrin family and found that EphA4 was deactivated in α-syn^−/−^ mice. Down-regulation of NCK2 and deactivation of Eph4 can be reversed by overexpression of α-syn, indicating that NCK2-Eph A4 signaling pathway was responsible, at least partially responsible for this α-syn mediated aberrant projection.

It was reported that NCK2 belongs to the adaptor protein family. It binds to the phosphorylated tyrosine residue of the upstream signal protein via SH2 domain and binds to the proline rich domain of the downstream signal protein via SH3 domain [43]. The junction protein achieves signal transmission between upstream and downstream signaling proteins through this connection. In the present study, we found that NCK2 was associated with Eph A4, and Rho A. Nonetheless, Rho A activity was lower in α-syn deficient mice, indicating that NCK2 binds Eph A4 and RhoA, further activating Rho A. Rho A, as a mammalian gene homolog of the Ras family, is a member of the Rho subfamily of the small molecular weight GTPase superfamily. Rho is regulated by various cytokines; GTP enzyme activating proteins (GAPs) and GDP dissociation inhibitors (GDIs) are Rho in-activators. Activators can enable Rho A to release GDP and combine with GTP; GAPs can activate the GTP enzyme activity of Rho A molecules themselves. Hydrolyze GTP into GDP; GDIs can suppress the transition between Rho GDP and Rho GTP states. Under the regulatory action of the above mentioned molecules, Rho completes the transition between the two states to achieve the "molecular switching" effect in its signal transduction process [44]. Activated Rho GTP combines with its downstream targets eg. Rho kinase (ROCK) to activate the latter, causing recombination of Cytoskeleton [45].

In the physiological conditions, Eph A4 receptors were activated by their ligands Ephrin A4. NCK2, as an adapor, binds to Eph A4 and Rho A-GDP. RhoA-GDP was phosphorylated to be Rho A-GTP (the active form of Rho A). The Rho A-GTP further activated its downstream signaling pathway and cytosol skeleton was reorganized. NCK2 protein level was regulated by α-syn. Hence, NCK2 protein level is not sufficient in α-syn/Snac KO mice. That is the molecular mechanism why OSNs projected aberrantly and resulted in mice hyposmia.

There are still some limitation present in the current study. Eg. the mechanism that α-syn regulates NCK2 protein level is still unclear. But it was reported that α-syn is abundant in nucleus and it regulate several other genes transcription as a transcriptional factor [46, 47]. Hence, it is possible that α-syn transcriptionally modulate NCK2 gene expression and protein translation accordingly. However, further experimental research is required to elucidate. In addition, in the process of α-syn deficiency induced hyposmia, we cannot rule out the possibility that a-syn also participates in olfactory regulation by influencing other factors except for NCK2. Also, we are using α-syn whole body KO model which cannot rule out the interference of changes in other tissues or organs on the sense of smell. These need to be explored through more research in the future.

## Conclusions

**Fig. 10:**
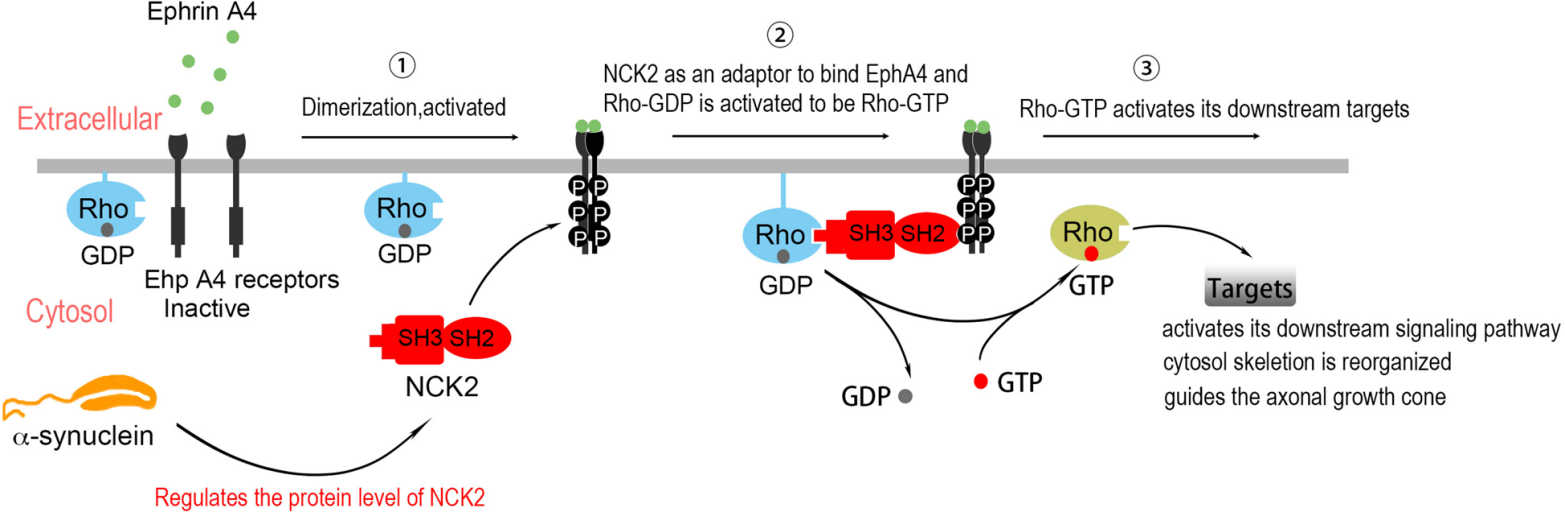
α-syn modulates NCK2-EphA4/RhoA pathway by regulating NCK2 protein level

Our study suggests that α-syn regulates NCK2 protein level and controls the precise axon guidance of olfactory neurons via NCK2-EphA4/RhoA pathway. NCK2 modulates EphA4/Rho A signaling pathway by interacting with EphA4 and RhoA as an adaptor (Fig. 10).

## Supplementary Information

Supplementary Material 1.

## Data Availability

The data that support the findings of this study are available from the corresponding author upon reasonable request.

## Abbreviations

PD: Parkinson’s disease
α-syn: α-Synuclein
NAC: Non-amyloid-b component
SNAC ^−^^/^^−^: Snac/α-synuclein knockout
WT: Wild-type
KO: Knockout
OB: Olfactory bulb
SCX: Strong cation exchange chromatography
LC: Liquid chromatography
IDA: Information dependent acquisition
CID: Collision-induced dissociation
Crt: Cortex
Cere: Cerebellum
OE: Olfactory epithelium
Str: Striatum
SN: Substantia nigra
OR: Olfactory receptor
RTK: Receptor tyrosine kinase
