# Supplementary material for: A novel function for α-synuclein as a regulator of NCK2 in olfactory bulb: implications for its role in olfaction
Source: Cell Biosci. 2024 Nov 14;14:139. doi: 10.1186/s13578-024-01313-6 (PMC11566155; doi:10.1186/s13578-024-01313-6)
Supplement: Supplementary file 1 — Supplementary Material 1. [file 13578_2024_1313_MOESM1_ESM.pdf]

A full unedited gel of Fig. 1A

1 2 3 4 5 6

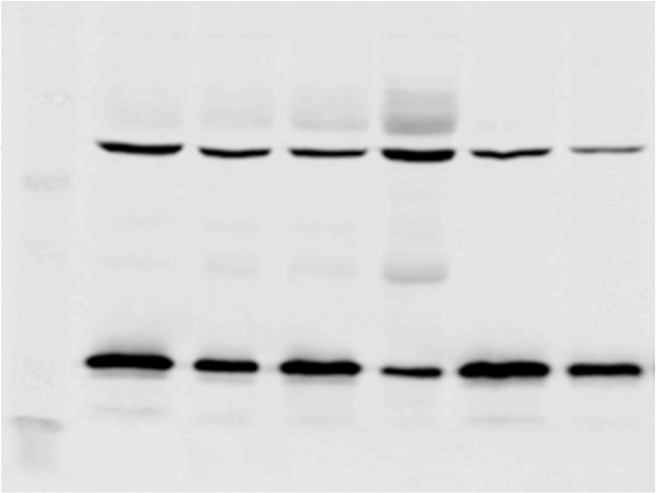

1. sample of Cortex
2. sample of Cerebellum
3. sample of OB
4. sample of OE
5. sample of Striatum
6. sample of SN

B full unedited gel of Fig. 3C

1 2 3 4

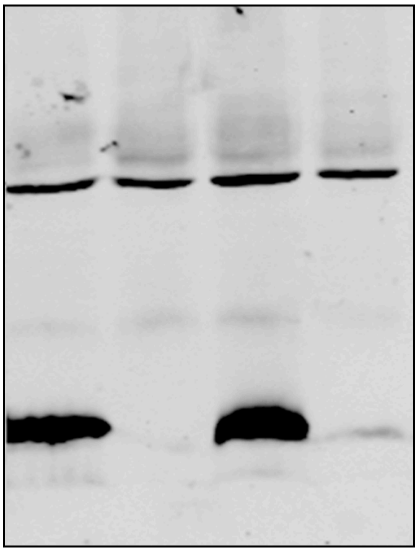

1. sample of wild-type mouse 1
2. sample of a-synuclein knockout mouse 2
3. sample of wild-type mouse 1
4. sample of a-synuclein knockout mouse 2

b-actin (top bands), a-synuclein (bottom bands)

C full unedited gel of Fig. 4C

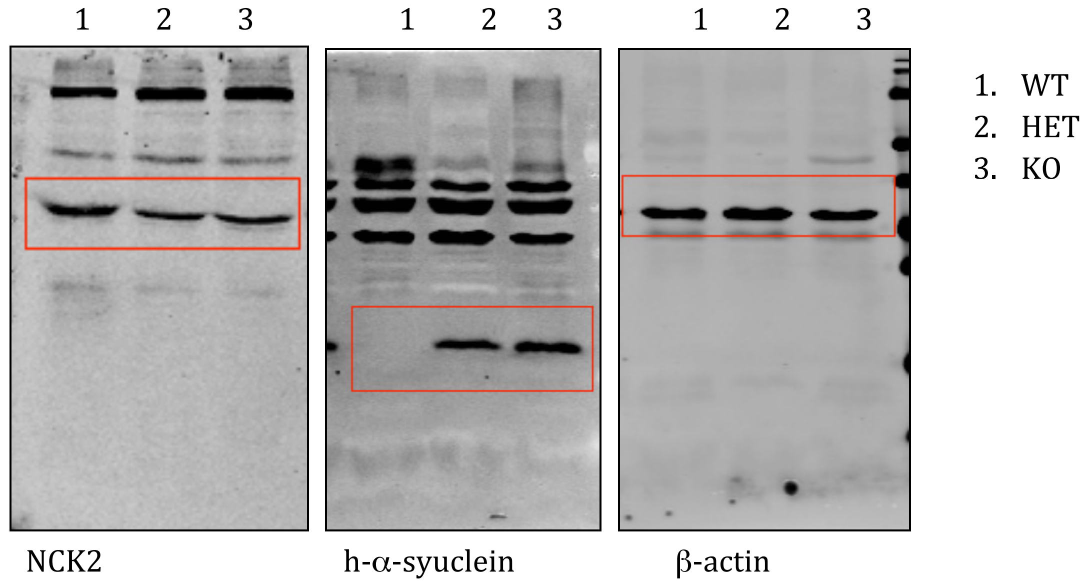

D full unedited gel of Fig. 4D

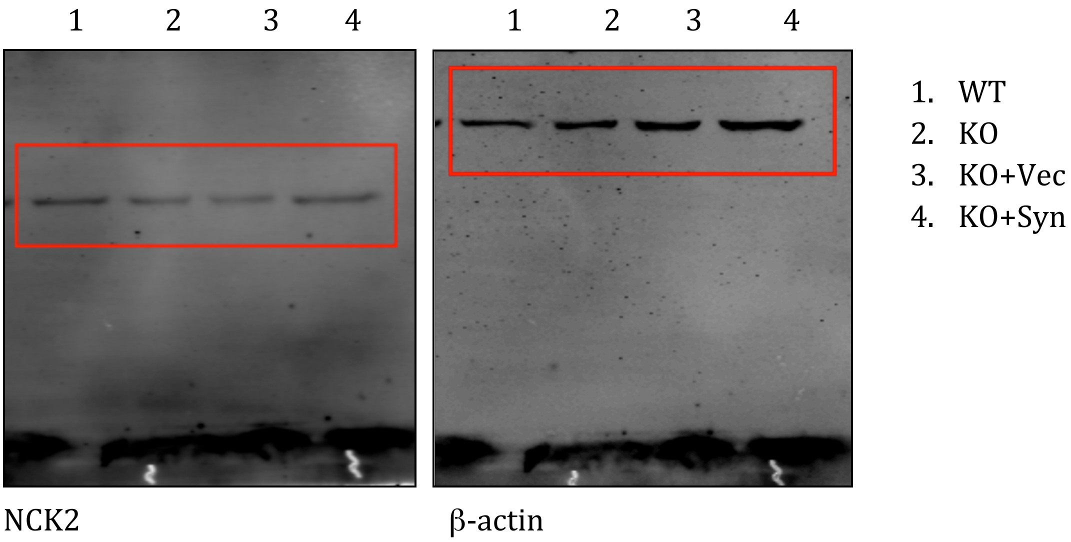

E full unedited gel of Fig. 4E

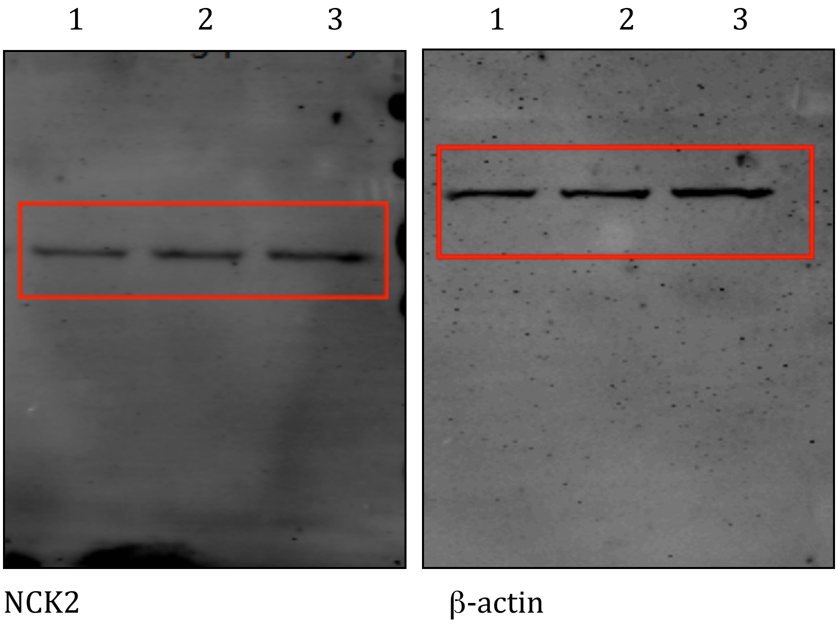

1. WT
2. WT+GFP
3. WT+Syn

F full unedited gel of Fig. 6B

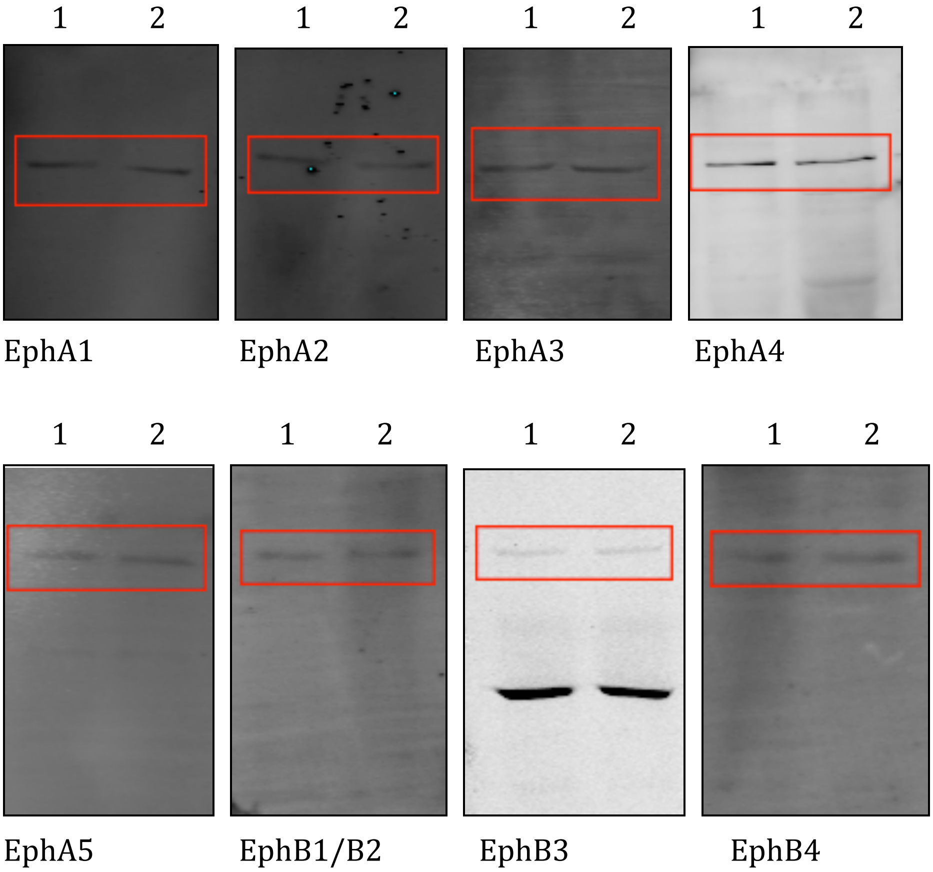

1 2 1 2 1 2 1 2

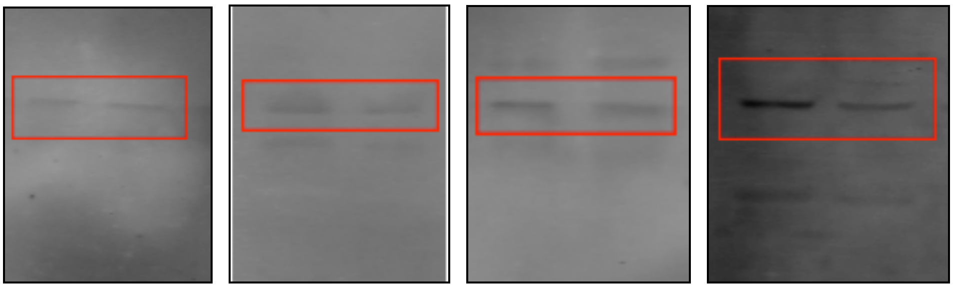

pEphA1 pEphA2 pEphA3 pEphA4

1 2 1 2 1 2 1 2

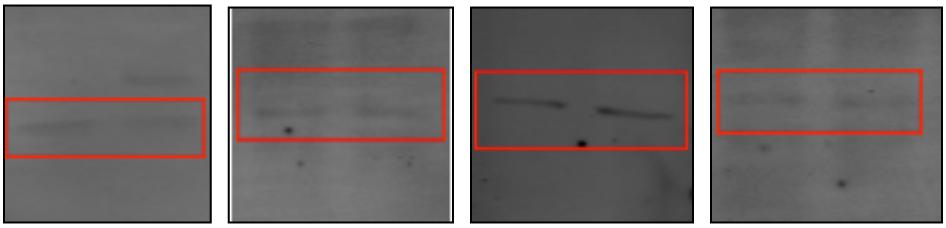

pEphA5 pEphB1/B2 pEphB3 pEphB4

1 2 1 2 1 2

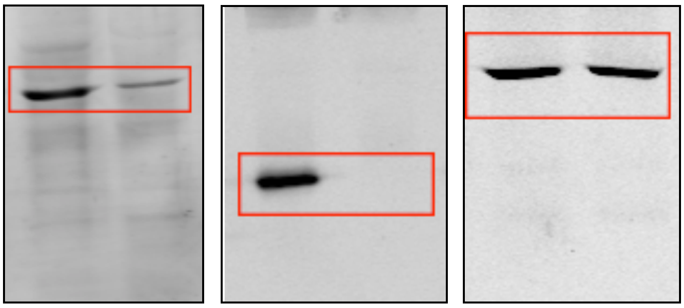

NCK2 α-Synuclein β-actin

1. WT
2. KO

G full unedited gel of Fig. 6D

1 2 3 4 5 6 7 8 9 1 2 3 4 5 6 7 8 9

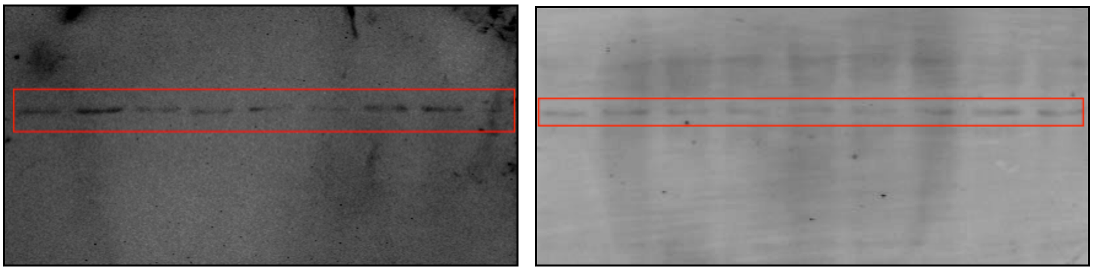

pEphA4 EphA4

1 2 3 4 5 6 7 8 9 1 2 3 4 5 6 7 8 9

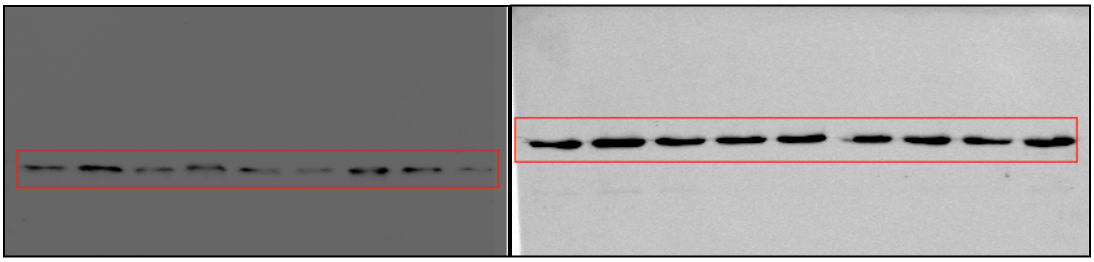

NCK2 β-actin

1. Scramble 24h
2. siRNA 1# 24h
3. siRNA 2# 24h
4. Scramble 48h
5. siRNA 1# 48h
6. siRNA 2# 48h
7. Scramble 72h
8. siRNA 1# 72h
9. siRNA 2# 72h

H full unedited gel of Fig. 6G

1 2 3 4 5 6 7 8 1 2 3 4 5 6 7 8

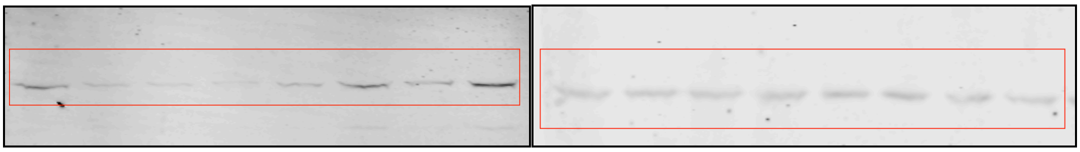

pEphA4 EphA4

1 2 3 4 5 6 7 8 1 2 3 4 5 6 7 8

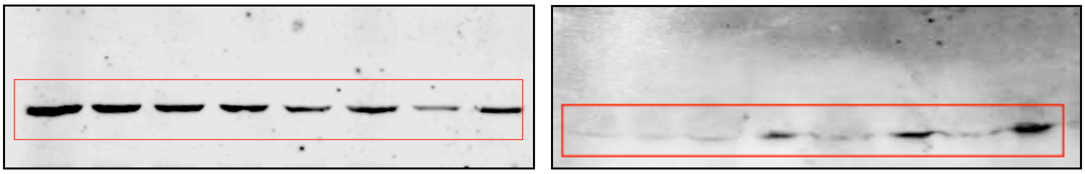

NCK2 h-α-syn

1 2 3 4 5 6 7 8

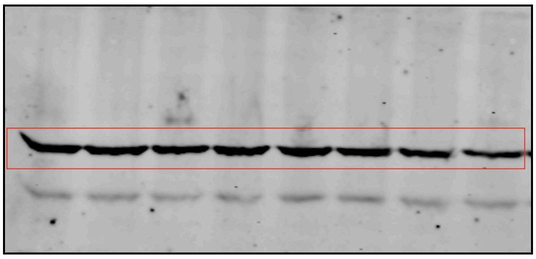

β-actin

1. WT
2. KO
3. KO+EV 24h
4. KO+α-Syn 24h
5. KO+EV 48h
6. KO+α-Syn 48h
7. KO+EV 72h
8. KO+α-Syn 72h

I full unedited gel of Fig. 8C

1 2 3 4 5 6

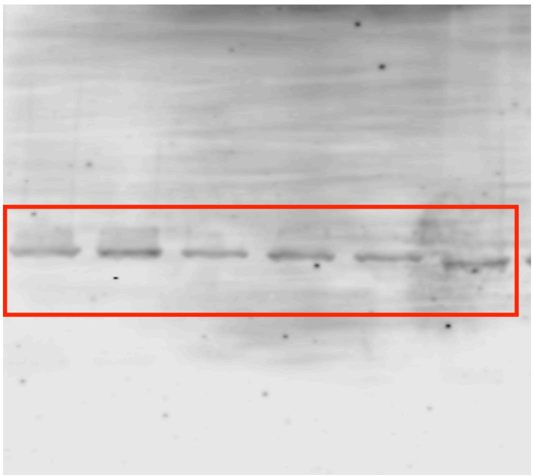

pEphA4

1 2 3 4 5 6

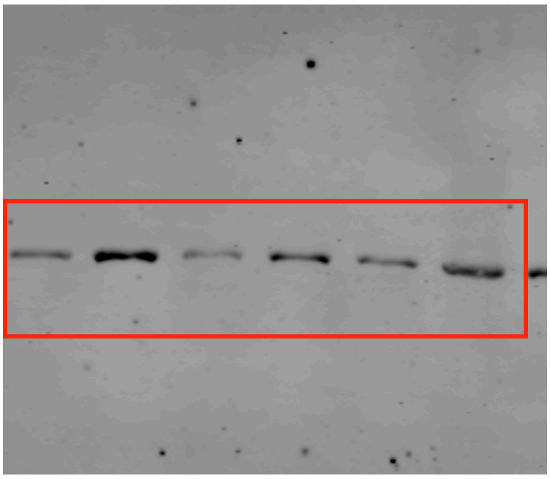

NCK2

1 2 3 4 5 6

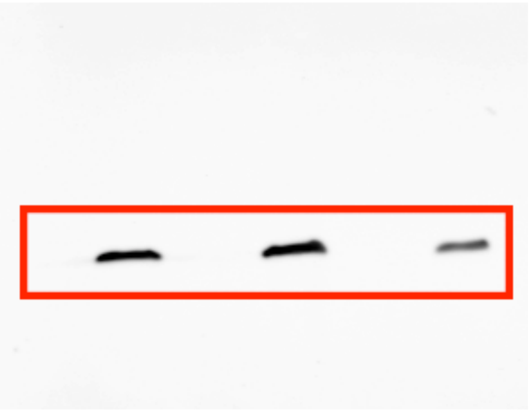

α-synuclein

1 2 3 4 5 6

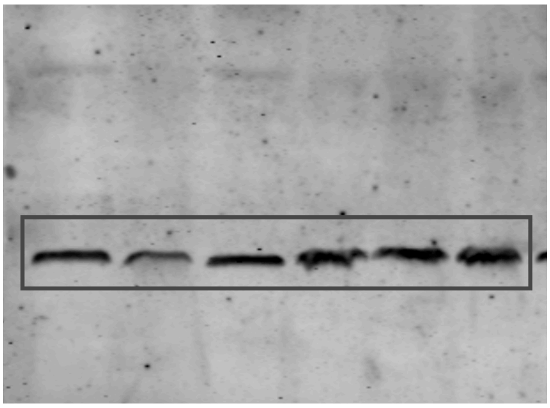

β-actin

1. Ctr1 2. Rsc1 3. Ctr2 4. Rsc2 5. Ctr3 6. Rsc3

J full unedited gel of Fig. 9A

1 2 3

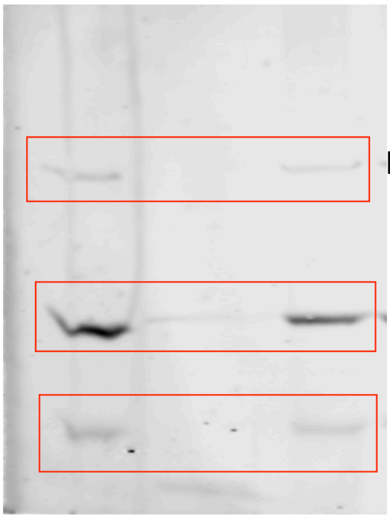

EphA4

NCK2

RhoA

1. Input
2. IP with IgG
3. IP with NCK2

K full unedited gel of Fig. 9B

1 2 3 4 5 6

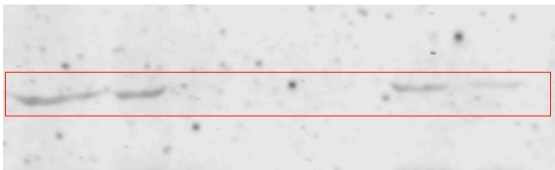

EphA4

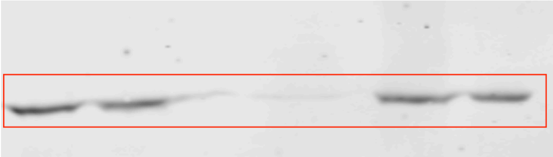

NCK2

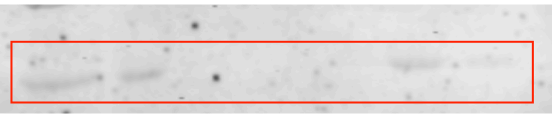

RhoA

1. Input: WT
2. Input: KO
3. IP with IgG: WT
4. IP with IgG: KO
5. IP with NCK2: WT
6. IP with NCK2: KO

L full unedited gel of Fig. 9C

1 2 3 4 5

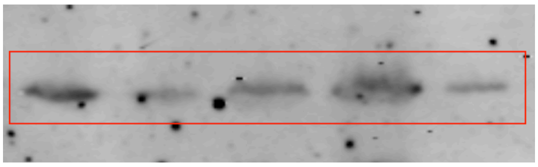

1 2 3 4 5

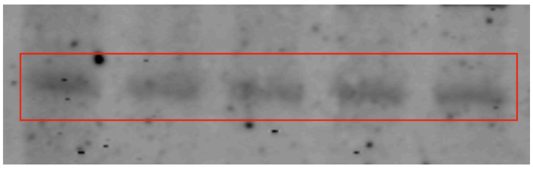

active Rho A

1 2 3 4 5

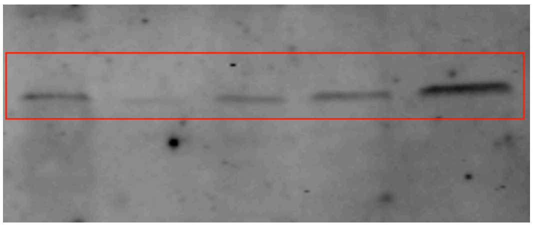

Rho A

1 2 3 4 5

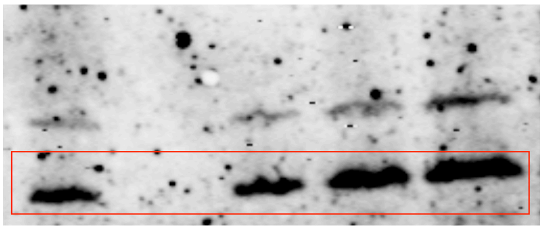

h-α-Syn

NCK2

1 2 3 4 5

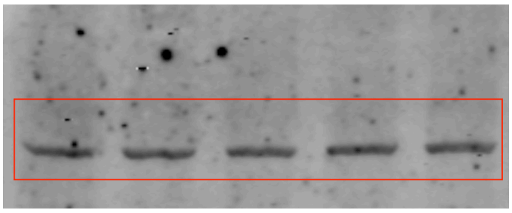

β-actin

1. WT
2. KO
3. KO+LV-Syn lower concentration
4. KO+LV-Syn higher concentration
5. KO+LV-Syn highest concentration
